# Real-world evaluation of health-related quality of life in patients with diffuse large B-cell lymphoma based on a multinational survey

**DOI:** 10.3389/fonc.2024.1402992

**Published:** 2024-06-24

**Authors:** P. Connor Johnson, Abigail Bailey, Qiufei Ma, Neil Milloy, Jake Butcher, Isaac Sanderson, Sarah Weatherby, Rachael Meadows, Ruben G. W. Quek

**Affiliations:** ^1^ Cancer Center, Massachusetts General Hospital, Boston, MA, United States; ^2^ Adelphi Real World, Bollington, United Kingdom; ^3^ Health Economics and Outcomes Research, Regeneron Pharmaceuticals Inc., Tarrytown, NY, United States

**Keywords:** diffuse large B-cell lymphoma, disease burden, quality of life, cross-sectional study, patient reported outcome measures

## Abstract

**Background:**

Real-world health-related quality of life (HRQoL) data in patients with diffuse large B-cell lymphoma (DLBCL) are scarce. This study is to compare patient-reported outcomes in patients with DLBCL across therapy lines and countries.

**Methods:**

Data were derived from the Adelphi DLBCL Disease Specific Programme™ from January 2021 to May 2021, a survey of physicians and their DLBCL patients in France, Germany, Italy, Spain, United Kingdom (UK), and the United States (US).

**Results:**

Overall, analysis was conducted on 441 patients with DLBCL across Europe and the US (mean age 64.6 years, 64% male); 68% had an Ann Arbor stage III and 69% had an Eastern Cooperative Oncology Group Performance Status of 0 to 1. The mean overall GHS/QoL was 54.1; patients on their 3L+ therapy had a lower mean GHS/QoL compared with patients on 1L/2L (*P* = 0.0033). Further to this, mean EQ-5D-5L utility score was reduced from 0.73 for patients on 1L therapy to 0.66 for patients on 3L+ therapies (*P* = 0.0149). Mean percentages of impairment while working and overall work impairment were lower for patients receiving 3L+ therapy (12.5% and 17.7%; respectively) than those on 1L therapy (35.6% and 33.8%; respectively). When comparing region, patients in the US had significantly better scores for all functioning and symptomatic scales (per EORTC QLQ-C30) and work impairment (per WPAI) vs. patients with DLBCL in Europe. WPAI scores indicate that the overall activity impairment in the US was 36.6% and in Europe ranged from 42.4% in the UK to 54.9% in Germany. Mean EQ-5D-5L utility score for the US was 0.80, compared to 0.60 – 0.80 across the countries in Europe. Regression analysis showed patients who relapsed after more than one year of treatment were associated with better patient reported outcomes than those who relapse after less than one year.

**Conclusion:**

Patient-reported outcomes of DLBCL patients remain poor and patients continue to experience considerable morbidity.

## Lay abstract

There is little information about the effects of diffuse large B-cell lymphoma (DLBCL) and treatments on quality of life (QoL) as assessed by patients. Information from patients taken from a large real-world survey of physicians and their DLBCL patients in France, Germany, Italy, Spain, UK, and the US showed that the QoL of patients worsened in later lines of treatment, after responding poorly to the previous treatment; symptoms also increased. QoL, including ability to work, was considerably better for patients in the US than in Europe, and for patients who deteriorated in health after a period of improvement after one year on treatment than patients who relapsed before one year. DLBCL patients, especially those on their third (or more) therapy, live with considerable morbidity despite receiving treatment.

## 1 Introduction

Non-Hodgkin lymphoma (NHL) is ranked as the fifth to ninth common cancer among most countries globally (1) and the seventh most common cancer in the United States (US) alone, while accounting for the sixth highest mortality among cancers in the US (2). The most common NHL subtype is diffuse large B-cell lymphoma (DLBCL) that accounts for 30%–58% of total NHL (3). DLBCL has a variable prognosis, depending on age, stage, extra-nodal sites, serum lactate dehydrogenase level, performance status and response to therapy (3).

A common first-line treatment is multiagent chemo-immunotherapy, which is potentially curative (3) but carries a significant risk of toxicities and healthcare utilization (4–6). Response to initial therapy is inadequate in 30–40% of patients (7). Treatments in the relapsed/refractory setting include chimeric antigen receptor T-cell (CAR-T) therapy and salvage chemotherapy followed by autologous stem cell transplant (ASCT) (4). Both therapies are intensive treatments with potentially significant toxicities. CAR-T therapy carries risk of cytokine release syndrome and neurologic toxicity (8) and ASCT involves intensive multiagent chemotherapy and multiweek hospitalization. Moreover, many patients either are not eligible for or do not respond to these therapies, and ultimately progress and die of their disease (9–13).

Significant differences have been noted between countries in the incidence and mortality of NHL (14), but little data currently exists on how the patient experience differs between different countries. Given the differences in healthcare systems and guidelines between different countries, patients may experience their disease in significantly different ways and may have different outcomes.

Evaluation of patient-reported outcomes (PRO) contributes to more informed and individualized treatment decisions in daily clinical practice and more accurate data on the disease impact experienced by the patient (15). Routine patient-reported symptom monitoring in adults with metastatic solid tumors has been associated with improved QoL and survival (16). However, there is both a paucity of real-world PRO data in DLBCL to facilitate patient evaluation (17), in spite of the toxicities associated with available therapies and associated potential for impaired quality of life (QoL). Notably, despite there being several novel therapies in development or in late-stage clinical testing, there is a lack of comprehensive PRO data that could help clinicians understand where unmet needs exist and guide treatment discussions.

To address this lack of data, this analysis of data from a multinational real-world clinical population aimed to explore the impact of DLBCL on patient QoL across the US and five European countries, in order to investigate the line of treatment effects and potential differences across countries.

## 2 Materials and methods

### 2.1 Survey design

Real-world data was drawn from the Adelphi DLBCL Disease Specific Programme (DSP)™ database, a multinational, cross-sectional survey with elements of retrospective data analysis of physicians and their adult patients conducted in the US and five European countries (France, Germany, Italy, Spain, and the UK), conducted between January 2021 and May 2021. The survey was comprised of an online physician survey, and online medical record data abstraction patient record form completed by physicians, and a voluntary patient self-completion form. Physicians and their patients were recruited from a geographically diverse sample on a voluntary basis. The DSP methodology has been previously published and validated (18–22) and surveys have been implemented globally across many different disease areas.

Physicians involved with the management and treatment of patients with DLBCL were requested to complete surveys on up to six consecutive consulting patients with DLBCL who met the patient eligibility criteria. The six patients included one patient on first-line therapy (1L), three patients on second-line therapy (2L), and two patients on third-line therapy and beyond (3L+) or receiving best supportive care following 2L therapy.

Physicians could only report data they had on hand at the time of the consultation, thus representing the available evidence they have when making routine clinical treatment and other management decisions. Data included patient demographics and clinical characteristics, treatment patterns, and clinical outcomes.

#### 2.1.1 Physicians

Physicians were eligible to participate in this survey if they were medical oncologists, hematologists or hem-oncologists. Physicians had to be personally responsible for treatment decisions and management of patients with DLBCL and make treatment decisions for at least four patients with DLBCL in a typical month.

#### 2.1.2 Patients

Patients were recruited on a voluntary basis by their physicians. Patients were eligible for inclusion if they were over the age of 18 years at the time of data collection, had a physician-confirmed diagnosis of DLBCL, and were receiving active drug treatment for DLBCL at time of data collection or receiving best supportive care after completing 2L drug treatment. Patients were excluded if they were participating in a clinical trial. Steroid monotherapy was not considered to be an active drug treatment.

Patients were invited to complete a patient self-completion form detailing their perspective on patient demographics, current condition, patient reported outcomes, symptoms and perceptions regarding treatment. To reflect the burden of DLBCL, the patient-reported form also included questions on the emotional and physical impact of DLBCL using the EuroQol EQ-5D questionnaire (EQ-5D-5L) (1). Disease specific QoL was assessed using the EORTC QLQ-C30 questionnaire (23–26). Patient impairment was assessed by asking patients to assess the impact of DLBCL on their work and general activity with the Work Productivity and Activity Impairment questionnaire (WPAI) (27).

### 2.2 Survey measures

The EORTC QLQ-C30 questionnaire comprises a global health status (GHS)/HRQoL scale, five functional scales (physical, role, emotional, cognitive, and social functioning), eight symptom scales (fatigue, nausea/vomiting, pain, dyspnea, insomnia, appetite loss, constipation, and diarrhea) and a financial difficulties scale. Scale scores are linearly converted to range from 0 to 100. For the functioning and GHS/HRQoL scales, higher scores indicate better functioning; for the symptom and financial difficulties scales, higher scores indicate worse symptom burden and greater financial difficulties, respectively (24, 25).

The EQ-5D-5L (1) comprises five dimensions, including mobility, self-care, usual activities, pain/discomfort, and anxiety/depression, to assess health status and produce a utility score. The EQ-5D-5L utility score and domain scores range from -0.281 to 1.00, where higher scores indicate better QoL, and a score less than 0 indicates a QoL worse than death. The EQ visual analogue score (VAS) ranges from 0 (worst health imaginable) to 100 (best health imaginable) (1, 28). The 3L Crosswalk method was used to score the EQ-5D-5L, as this is the method recommended in the UK (29). To enable comparisons across countries, all patients were scored using this method irrespective of which country they were from.

The WPAI is a questionnaire for assessing work and activity impairments of patients. In this study, a specific health problems version of the WPAI was used to measure the specific impact of the patient’s DLBCL (27, 30). The WPAI questionnaire comprises four dimensions. Absenteeism is evaluated as percentage of work time missed due to DLBCL, with a higher percentage indicating more work time missed. Presenteeism is evaluated as percentage impairment while working due to DLBCL, with a higher percentage indicating more impairment at work due to DLBCL. Work productivity loss is evaluated as overall work impairment due to DLBCL, with a higher percentage indicating more impairment and work time missed to DLBCL. These were answered by all patients in part-time or full-time employment. The section of the WPAI on activity impairment (overall activity impairment due to DLBCL – a higher percentage indicating higher impairment carrying out normal daily activities excluding employment) was answered by patients regardless of employment status (27).

PRO outcomes were compared to an NHL reference population to better understand the impact of DLBCL relative to NHL patient populations. The reference population consists of 267 patients with NHL, of which the most common subtype of disease is DLBCL (31).

### 2.3 Statistical analysis

Descriptive analyses were derived using the software package IBM SPSS Data Collection Survey Reporter Version 7, while all inferential analyses were conducted using Stata v17 (32). Continuous variables were described as means and standard deviations (SD) and categorical variables were described as numbers and percentages.

Data were analyzed by country (France, Germany, Italy, Spain, UK, US) and region (Europe, US). Missing data were not imputed; therefore, the base of patients for analysis could vary from variable to variable and was reported separately for each analysis.

Validated NHL reference scores (25), which summarize patient burden for patients with NHL, were compared against the DSP patient EORTC QLQ-C30 scores using a t-test. DSP patient EQ-5D utility and EQ VAS scores were evaluated using minimally important differences (MID) assessed for patients with cancer, including lymphoma (33). The MID was 0.08 for EQ-5D UK-index scores, and 7 for EQ VAS scores.

We performed descriptive and bivariate analyses to compare PROs (EORTC QLQ-C30, EQ-5D-5L, and WPAI) across line of therapy (1L vs. 2L vs. 3L+, and 1L/2L vs. 3L+). ANOVA tests were performed to assess for differences in means between lines of therapy (1L vs. 2L vs. 3L+). Where significant differences were found, t-tests were performed to assess if differences were attributable to earlier lines compared with later lines of therapy (1L/2L vs. 3L+). Chi square tests were conducted to compare the differences in proportion of adverse events, across line of therapy (1L vs. 2L vs. 3L+). No adjustments for multiplicity were conducted.

Linear regressions were performed to assess the impact of covariates on EORTC QLQ-C30, EQ-5D-5L utility, EQ VAS, and WPAI scores. Based on their known association with outcomes in DLBCL, chosen covariates in this regression were lines of therapy (1L/2L vs. 3L+) (34), time to relapse (35), International Prognostic Index score (IPI; 0–4) (36), double exposure (has received a CD20 antibody and an alkylating agent) (37), receiving an ASCT (34), CAR-T cell therapy (38, 39) and disease state (double hit, double expressor, none of these) (40). Double hit/expressor (translocation of *MYC* with *BCL-2* or *BCL-6* rearrangement/overexpression of *MYC*, *BCL-2* or *BCL-6*) and triple hit/expressor (translocation of *MYC* with *BCL-2* and *BCL-6* rearrangement/overexpression of *MYC*, *BCL-2* and *BCL-6*) were all captured as individual items in the survey and grouped accordingly owing to similarities in genetic profile. Consequently, reference to the term double hit/expressor throughout the manuscript also includes triple hit/exposure definitions. The only continuous covariate in the regression models was time to relapse; the remaining covariates in the regression were all categorical. For these analyses, a p*-*value of <0.05 was considered statistically significant.

### 2.4 Ethics

This analysis used data that already exist in a secondary electronic database. Physician and patient data were pseudo-anonymized. A code was assigned when data were collected. Upon receipt by Adelphi Real World, data were pseudo-anonymized again to mitigate against tracing them back to the individual. Data were aggregated before being shared with the subscriber and/or for publication. The identity of the physicians was blinded, and no patient identifiers were collected. The study protocol was approved by the Western Institutional Review Board (Reference: AG-8980). Data were collected according to the European Pharmaceutical Marketing Research Association (EphMRA) guidelines and as such did not require Institutional Review Board/Independent Ethics Committee approval (41). Before participating, patients who provided data directly gave written informed consent using a tick box, which stated they had read and understood the conditions of the patient self-completion form; and did not provide any self-identifying data. Patient-reported forms were completed by the patient independently from their physician and returned in a sealed envelope ensuring the patient’s responses were kept confidential and ensuring future physician-patient interactions were not influenced from their physician. There was no restriction with regards to treatment received.

The DSP was performed in full accordance with relevant legislation at the time of data collection, including the Health Information Technology for Economic and Clinical Health Act legislation (42).

## 3 Results

All results discussed below regarding disease and symptom burden and adverse events are statistically significant (p<0.05), unless stated otherwise.

### 3.1 Physician and patient sample populations

A total of 239 oncologists/hematologists/hem-oncologists were recruited. Physicians completed a patient record form for a total of 1,418 eligible patients, from France, Germany, Italy, Spain, the UK, and the US. Of these patients, a total of 441 patients completed a patient self-completion form, equivalent to a 31% response rate (**Table 1**).

**Table 1:** Physician and patient sample populations.

	Total	France	Germany	Italy	Spain	UK	US
Physicians, n (%)	239	46 (19.2)	40 (16.7)	40 (16.7)	41 (17.2)	32 (13.4)	40 (16.7)
Physician-completed ePRF, n (%)	1418	254 (17.9)	240 (16.9)	240 (16.9)	241 (17.0)	199 (14.0)	244 (17.2)
Patient-completed PSC, n (%)	441	80 (18.1)	150 (34.0)	54 (12.2)	43 (9.8)	34 (7.7)	80 (18.1)

ePRF, electronic patient record form; PSC, patient self-completion form; United Kingdom, UK; United States, US.

### 3.2 Patient demographics and clinical characteristics

Of the 441 patients with DLBCL that completed PSCs, the mean (SD) age was 64.6 (12.0) years, 64% were male, and 19% were working full/part-time at data collection (**Table 2**). Patients age ranged from mean (SD) 68.0 (11.3) in France, to 60.4 (12.2) in the UK; while the mean age in the US was 62.6 years. The percentage of males in the sample was 71% in the UK, and 56% in the US. Less than one-third of European patients were working full/part-time (range: 5% Spain; 31% Italy) and ≥93% had public healthcare insurance (excluding France: a mix of public/private healthcare 61%); whereas in the US, 39% were working full/part-time and 80% had private healthcare insurance.

**Table 2:** Demographics and clinical characteristics of patients with DLBCL.

Characteristic	Total (n=441)	France (n=80)	Germany (n=150)	Italy (n=54)	Spain (n=43)	UK (n=34)	US (n=80)
Age, years*							
Mean (SD)	64.6 (12.0)	68.0 (11.5)	66.3 (11.3)	62.2 (13.8)	62.2 (13.7)	60.4 (12.2)	62.6 (12.5)
Median (min, max)	67.0 (23, 88)	71.5 (32, 88)	69.0 (32, 84)	65.0 (23, 84)	62.0 (25, 87)	61.0 (27, 76)	65.5 (28, 81)
Gender, n (%)							
Male	282 (64)	52 (65)	104 (69)	32 (59)	25 (58)	24 (71)	45 (56)
Female	159 (36)	28 (35)	46 (31)	22 (41)	18 (42)	10 (29)	35 (44)
Employment status*, n (%)							
Working full time	72 (16)	7 (9)	16 (11)	13 (24)	2 (5)	7 (21)	27 (34)
Working part time	13 (3)	0 (0)	2 (1)	4 (7)	0 (0)	3 (9)	4 (5)
On long term sick leave	57 (13)	11 (14)	17 (11)	7 (13)	17 (40)	4 (12)	1 (1)
Homemaker	35 (8)	2 (3)	15 (10)	4 (7)	7 (16)	1 (3)	6 (8)
Student	2 (<1)	0 (0)	0 (0)	1 (2)	1 (2)	0 (0)	0 (0)
Retired	245 (56)	57 (71)	99 (66)	23 (43)	12 (28)	17 (50)	37 (46)
Unemployed	13 (3)	2 (3)	1 (1)	0 (0)	3 (7)	2 (6)	5 (6)
Don’t know	4 (1)	1 (1)	0 (0)	2 (4)	1 (2)	0 (0)	0 (0)
Health insurance type*, n (%)							
Private	72 (16)	0 (0)	8 (5)	0 (0)	0 (0)	0 (0)	64 (80)
Public	297 (67)	23 (29)	139 (93)	54 (100)	41 (95)	34 (100)	6 (8)
Mixed	64 (15)	49 (61)	3 (2)	0 (0)	2 (5)	0 (0)	10 (13)
Don’t know	8 (2)	8 (10)	0 (0)	0 (0)	0 (0)	0 (0)	0 (0)
Ann Arbor stage*^†^, n (%)							
Stage I	36 (8)	5 (6)	6 (4)	2 (4)	7 (16)	10 (29)	6 (8)
Stage II	105 (24)	12 (15)	48 (32)	20 (37)	3 (7)	4 (12)	18 (23)
Stage III	125 (28)	26 (33)	40 (27)	15 (28)	16 (37)	17 (50)	11 (14)
Stage IV	175 (40)	37 (46)	56 (37)	17 (31)	17 (40)	3 (9)	45 (56)
ECOG-PS*^‡^, n (%)							
0	90 (20)	18 (23)	14 (9)	21 (39)	10 (23)	11 (32)	16 (20)
1	218 (49)	39 (49)	78 (52)	18 (33)	17 (40)	20 (59)	46 (58)
2	108 (24)	16 (20)	50 (33)	12 (22)	13 (30)	3 (9)	14 (18)
3+	25 (6)	7 (9)	8 (5)	3 (6)	3 (7)	0 (0)	4 (5)
Disease status*, n (%)							
Disease progressing	31 (7)	3 (4)	9 (6)	9 (17)	5 (12)	0 (0)	5 (6)
Stable	146 (33)	26 (33)	46 (31)	18 (33)	6 (14)	12 (35)	38 (48)
Responding to treatment	264 (60)	51 (64)	95 (63)	27 (50)	32 (74)	22 (65)	37 (46)
Charlson Comorbidity Index*							
Mean (SD)	2.3 (0.80)	2.2 (0.58)	2.4 (0.86)	2.2 (0.49)	2.3 (1.13)	2.3 (1.25)	2.2 (0.53)
Median (min, max)	2.0 (2, 9)	2.0 (2, 5)	2.0 (2, 6)	2.0 (2, 4)	2.0 (2, 8)	2.0 (2, 9)	2.0 (2, 4)
Line of therapy*, n (%)							
First-line treatment	87 (20)	18 (23)	32 (21)	9 (17)	12 (28)	7 (21)	9 (11)
Second-line treatment	228 (52)	39 (49)	78 (52)	31 (57)	21 (49)	18 (53)	41 (51)
Third-line treatment or later, or best supportive care following second-line treatment	126 (29)	23 (29)	40 (27)	14 (26)	10 (23)	9 (26)	30 (38)

* At data collection.

^†^ Ann Arbor staging: stage I: involvement of a single lymph node region or of a single extralymphatic organ or site; stage II: involvement of two or more lymph node regions on the same side of the diaphragm or localized involvement of an extralymphatic organ or site; stage III: involvement of lymph node regions or structures on both sides of the diaphragm; stage IV: diffuse or disseminated involvement of one or more extralymphatic organs, or either: isolated extralymphatic organ involvement without adjacent regional lymph node involvement, but with disease in distant sites, OR involvement of the liver, bone marrow, pleura or cerebrospinal fluid.

^‡^ ECOG-PS score 0–1, Fully active, able to carry on all pre-disease performance without restriction/restricted in physically strenuous activity but ambulatory and able to carry out work of a light or sedentary nature, e.g., light house work, office work; ECOG-PS score ≥2, ambulatory and capable of all selfcare but unable to carry out any work activities, up and about more than 50% of waking hours/capable of only limited selfcare, confined to bed or chair more than 50% of waking hours/completely disabled, cannot carry on any selfcare, totally confined to bed or chair.

DLBCL, Diffuse large B-cell lymphoma; ECOG-PS, Eastern Cooperative Oncology Group Performance Status; SD, standard deviation; United Kingdom, UK; United States, US.

Two-thirds (68%) of patients had Ann Arbor stage III (28%) or stage IV (40%) disease and 20%, 49% and 24% of patients had an Eastern Cooperative Oncology Group Performance Status (ECOG-PS) score of 0, 1 and 2, respectively. In Europe, a variable proportion of patients had ECOG-PS scores of 0 (range: 9% Germany; 32% UK) and 2 (range: 9% UK; 33%, Germany) compared with US patients (range: 20% and 18%, respectively). Patients each had a mean Charlson comorbidity index (SD) of 2.3 (0.8). The proportion of patients with Ann Arbor stage III disease in Europe ranged from 27% in Germany to 50% in the UK and was 14% in the US. Ann Arbor stage IV disease in Europe ranged from 9% in the UK to 46% in France, and 56% in the US.

At data collection, 60% of patients were responding to treatment, 33% of patients had stable disease, and 7% had disease progression. In total the majority of all patients (52%) were receiving 2L therapy. Response to treatment was found in a greater proportion of patients in Europe (range: 50% Italy; 74% Spain) than in the US (46%). Reports of stable disease were ≤35% in Europe and 48% in the US, and disease progression was variable in Europe (range: 0% UK; 17% Italy) vs US (6%). Compared with European patients, a greater proportion of US patients were receiving 3L+ treatment (US 38%; Europe range: 23% Spain; 29% France) and correspondingly, smaller proportion were receiving 1L (11% US; Europe range: 17% Italy; 28% Spain).

### 3.3 Disease burden and PROs

#### 3.3.1 EORTC DLBCL DSP vs NHL reference value mean scores for all lines of therapy combined

Mean EORTC QLQ-C30 functioning scale scores of the DSP DLBCL population were higher when compared with NHL reference values with regards to role functioning (61.7 vs. 57.3), cognitive functioning, and social functioning (64.4 vs. 60.4) (**Table 3**). Mean EORTC QLQ-C30 symptom scale scores were higher (i.e., worse) than NHL reference values with regard to nausea and vomiting (20.0 vs. 10.0), pain (27.3 vs. 24.5), dyspnea (25.3 vs. 16.9), appetite loss (35.3 vs. 19.9), and diarrhea (12.3 vs. 9.5).

**Table 3:** Bivariate analysis comparing EORTC QLQ-C30 scores for patients with DLBCL vs NHL reference values*^†^.

EORTC QLQ-C30	DLBCL DSP^TM^ Data results	NHL reference value*^†^		p value (DLBCL DSP^TM^ data vs NHL reference values) (TT)
Functional scores				
**Global health status (n=441),** mean (SD)	54.1 (19.1)	56.1 (27.1)		0.032
**Physical functioning (n=441),** mean (SD)	71.7 (21.6)	N/A^‡^		N/A^†^
**Role functioning (n=441),** mean (SD)	61.7 (28.0)	57.3 (36.2)		0.001
**Emotional functioning (n=441),** mean (SD)	67.7 (23.1)	66.8 (25.2)		0.413
**Cognitive functioning (n=441),** mean (SD)	75.8 (23.5)	68.5 (30.8)		<0.001
**Social functioning (n=440),** mean (SD)	64.4 (27.6)	60.4 (34.1)		0.003
Symptom scores				
**Fatigue (n=441),** mean (SD)	41.5 (23.7)		41.9 (28.9)	0.738
**Nausea & vomiting (n=441),** mean (SD)	20.0 (22.2)		10 (28.9)	<0.001
**Pain (n=441),** mean (SD)	27.3 (24.8)		24.5 (30.1)	0.020
**Dyspnea (n=441),** mean (SD)	25.3 (25.2)		16.9 (24.6)	<0.001
**Insomnia (n=439),** mean (SD)	30.3 (27.5)		30.7 (31.5)	0.759
**Appetite loss (n=440),** mean (SD)	35.3 (27.9)		19.9 (29.4)	<0.001
**Constipation (n=439),** mean (SD)	17.6 (24.4)		18.2 (27.8)	0.617
**Diarrhea (n=439),** mean (SD)	12.3 (18.7)		9.5 (19.1)	0.002
**Financial difficulties (n=439),** mean (SD)	17.0 (24.2)		17.8 (28.8)	0.494

*Scott NW, Fayers PM, Aaronson NK, et al. for the EORTC Quality of Life Group. The EORTC QLQ-C30 Reference values. EORTC Quality of Life Unit, Brussels, Belgium, 2008, page 205.

^†^ NHL reference values are not adjusted for age and sex; p values are for comparisons of mean values.

^‡^ There is no EORTC QLQ-C30 reference value for NHL physical functioning, as a previous version of this scale was used where reference values are unknown.

For the functioning scales and global QoL, higher scores indicate better functioning; For the symptom scales and financial difficulties, lower scores indicate better symptom burden and fewer financial difficulties, respectively.

DLBCL, Diffuse large B-cell lymphoma; EORTC QLQ-C30, European Organization for the Research and Treatment of Cancer Quality of Life Questionnaire Core 30; N/A, not available; SD, standard deviation; TT, T-test.

Compared with NHL reference values, analysis by region showed that patients in the US had higher scores for EORTC QLQ-C30 functioning scales and lower scores for both symptom scales and financial difficulties (**Table 4**), while European countries scored lower for EORTC QLQ-C30 functioning scales except cognitive functioning and symptom scales. Scores for financial difficulties were lower in France and Germany, and higher in Spain and the UK compared with NHL reference values.

**Table 4:** Disease burden of patients with DLBCL, by country: bivariate analysis.

Characteristic	Total (n=441)	France (n=80)	Germany (n=150)	Italy (n=54)	Spain (n=43)	UK (n=34)	US (n=80)	All- countries difference	Individual country vs. all other countries	Individual European country vs. all other European countries	Europe *vs*. US	NHL reference value	DLBCL vs. NHL reference value	Individual country vs. NHL reference value
								(AN) p value	(TT) p value*	(TT) p value*	(TT) p value		(TT) p value	(TT) p value
EORTC QLQ-C30														
**Global health status, n**	441	80	150	54	43	34	80	<0.0001	DE: 0.0003 UK: 0.0018 US: <0.0001	Overall: 0.0012 IT: 0.0002 UK: 0.0178	<0.0001	56.1 (58.3)	0.032	DE: <0.001 UK: <0.001 US: <0.001
Mean (SD)	54.1 (19.1)	51.7 (21.4)	49.6 (14.9)	59.3 (16.2)	52.2 (19.8)	44.4 (14.9)	67.5 (19.7)							
**Physical functioning, n**	441	80	150	54	43	34	80	<0.0001	DE: 0.0039 US: <0.0001	IT: 0.0088	<0.0001	N/A	N/A	
Mean (SD)	71.7 (21.6)	68.3 (21.9)	67.6 (20.9)	75.8 (19.4)	66.4 (24.3)	67.6 (18.2)	84.8 (18.1)							
**Role functioning, n**	441	80	150	54	43	34	80	<0.0001	FR: 0.0183 DE: 0.0001 US: <0.0001		<0.0001	57.3 (66.7)	0.001	US: <0.001
Mean (SD)	61.7 (28.0)	55.0 (31.0)	54.2 (25.9)	63.6 (27.1)	57.4 (26.8)	64.2 (23.3)	82.3 (21.3)							
**Emotional functioning, n**	441	80	150	54	43	34	80	<0.0001	ES: <0.0001 US: <0.0001	Overall: 0.0052 ES: 0.0004	<0.0001	66.8 (75)	0.413	ES: 0.001 US: <0.001
Mean (SD)	67.7 (23.1)	68.0 (24.0)	65.9 (20.6)	65.0 (22.2)	52.9 (25.8)	61.2 (20.2)	83.2 (18.4)							
**Cognitive functioning, n**	441	80	150	54	43	34	80	<0.0001	FR: 0.0462 ES: 0.0005 UK: 0.0061 US: <0.0001	DE: 0.0043 ES: 0.0098	<0.0001	68.5 (83.3)	<0.001	DE: <0.001 IT: 0.001 US: <0.001
Mean (SD)	75.8 (23.5)	71.0 (24.8)	77.0 (22.7)	75.3 (20.9)	64.0 (27.4)	65.2 (22.6)	89.4 (15.5)							
**Social functioning, n**	440	80	149	54	43	34	80	<0.0001	FR: 0.0021 ES: 0.0248 US: <0.0001		<0.0001	60.4 (66.7)	<0.0001 (AN)	US: <0.001
Mean (SD)	64.4 (27.6)	55.8 (29.9)	63.0 (25.3)	63.9 (28.2)	55.4 (28.6)	62.7 (22.1)	81.5 (23.6)							
**Fatigue, n**	441	80	150	54	43	34	80	<0.0001	FR: 0.0021 ES: 0.0478 US: <0.0001		<0.0001	41.9 (33.3)	0.738	FR: 0.017 US: <0.001
Mean (SD)	41.5 (23.7)	48.9 (25.6)	44.0 (23.1)	41.2 (21.8)	48.3 (26.1)	42.0 (16.3)	26.0 (18.5)							
**Nausea & vomiting, n**	441	80	150	54	43	34	80	0.0001	ES: 0.0021 US: 0.0002		0.0002	10 (0)	<0.001	FR: <0.001 DE: <0.001 IT: 0.002 ES: <0.001 UK: <0.001
Mean (SD)	20.0 (22.2)	22.9 (24.8)	19.2 (22.0)	18.2 (18.7)	29.8 (27.8)	26.5 (17.5)	11.7 (17.1)							
**Pain, n**	441	80	150	54	43	34	80	0.0008	ES: 0.0053 US: 0.0006	ES: 0.0245	0.0006	24.5 (16.7)	0.02	DE: 0.008 ES: 0.007 US: 0.017
Mean (SD)	27.2 (24.8)	26.5 (27.0)	29.8 (24.1)	23.1 (22.3)	37.2 (29.3)	31.9 (21.5)	18.8 (21.1)							
**Dyspnea, n**	441	80	150	54	43	34	80	0.0013	FR: 0.0196 US: <0.0001		<0.0001	16.9 (0)	<0.001	FR: <0.001 DE: <0.001 ES: 0.015 UK: 0.003
Mean (SD)	25.3 (25.2)	31.3 (25.1)	26.7 (25.6)	24.1 (22.8)	27.1 (26.5)	29.4 (22.9)	15.0 (23.7)							
**Insomnia, n**	439	80	149	54	43	33	80	<0.0001	ES: <0.0001 US: <0.0001	Overall 0.0011 FR: 0.0288 DE: 0.0179 IT: 0.0376 ES: 0.004	<0.0001	30.7 (33.3)	0.759	IT: 0.025 ES: <0.001 US: <0.001
Mean (SD)	30.3 (27.5)	37.1 (29.5)	30.6 (26.7)	29.6 (26.4)	47.3 (28.4)	34.3 (22.8)	12.5 (18.7)							
**Appetite loss, n**	440	80	149	54	43	34	80	<0.0001	FR: 0.0016 ES: 0.0004 US: <0.0001	Overall 0.0055 DE *vs* Europe 0.0366 ES *vs* Europe 0.0009	<0.0001	19.9 (0)	<0.001	FR: <0.001 DE: <0.001 IT: 0.025 ES: <0.001 UK: <0.001
Mean (SD)	35.3 (27.9)	44.2 (32.6)	34.0 (26.1)	30.9 (21.3)	49.6 (32.0)	39.2 (20.9)	22.5 (23.6)							
**Constipation, n**	439	79	150	54	42	34	80	<0.0001	FR: 0.0058 DE: 0.0034 ES: <0.0001 US: 0.0001	Overall <0.0001 DE: <0.0001 ES: <0.0001	<0.0001	18.2 (0	0.617	DE: 0.003 ES: <0.001 US: <0.001
Mean (SD)	17.6 (24.4)	24.5 (30.1)	12.9 (21.8)	19.1 (21.1)	35.7 (27.9)	20.6 (21.7)	7.9 (16.1)							
**Diarrhea, n**	439	80	150	53	42	34	80	0.0008	FR: 0.0368 UK: 0.0026 US: 0.0028	Overall 0.0218 DE: 0.0242 UK: 0.0097	0.0028	9.5 (0)	0.002	FR: 0.011 UK: 0.001
Mean (SD)	12.3 (18.7)	16.3 (23.1)	10.9 (17.1)	11.3 (15.9)	14.3 (18.3)	21.6 (19.9)	6.7 (16.3)							
**Financial difficulties, n**	439	80	149	54	43	33	80	<0.0001	FR: 0.007 DE: 0.0181 ES: <0.0001 UK: <0.0001	Overall <0.0001 FR: 0.002 DE: 0.0022 ES: 0.0001 UK: <0.0001	0.0951	17.8 (0)	0.494	FR: 0.002 DE: 0.008 ES: 0.007 UK: <0.001 US: 0.046
Mean (SD)	17.0 (24.2)	10.4 (20.9)	13.2 (20.8)	19.8 (23.8)	31.8 (32.5)	36.4 (24.1)	12.9 (21.5)							
EQ-5D-5L														
**EQ-5D Utility score (UK crosswalk), n**	439	78	150	54	43	34	80	<0.0001	DE: 0.0007 ES: 0.0424 US: <0.0001	UK: 0.0162	<0.0001			
Mean (SD)	0.70 (0.20)	0.70 (0.24)^‡^	0.70 (0.17)^§^	0.70 (0.22)^ǁ^	0.60 (0.20)^¶^	0.80 (0.15)^**^	0.80 (0.16)^††^							
**EQ VAS, n**	381	63	140	33	36	30	79	<0.0001	FR: <0.0001 DE: 0.0031 ES: 0.0093 US: <0.0001	Overall 0.0031 FR: 0.0168 UK: 0.0016	<0.0001			
Mean (SD)	64.6 (17.9)	56.2 (18.3)^‡^	61.0 (14.1)^§^	63.1 (14.4)^ǁ^	57.2 (20.8)^¶^	69.6 (16.5)^**^	79.6 (14.8)^††^							
WPAI^†^														
**% Work time missed due to problem, n**	66	1	12	14	3	7	29	0.0257	IT: 0.0291 US: 0.0019		0.0019			
Mean (SD)	28.4 (36.0)	0.0 (0.0)	31.3 (36.9)	46.9 (40.1)	36.2 (55.3)	49.8 (47.5)	13.2 (21.6)							
**% Impairment while working due to problem, n**	65	2	16	12	2	4	29	0.0004	DE: 0.024 IT: 0.0205 US: <0.0001		<0.0001			
Mean (SD)	30.3 (31.5)	55.0 (63.6)	45.6 (38.6)	49.2 (22.3)	50.0 (42.4)	22.5 (5.0)	12.1 (18.8)							
**% Overall work impairment due to problem, n**	55	1	10	10	2	4	28	0.0039	IT: 0.001 US: 0.0007		0.0007			
Mean (SD)	31.1 (27.5)	10.0 (0.0)	36.6 (26.0)	56.1 (26.6)	52.7 (39.2)	32.3 (4.8)	19.2 (23.5)							
**% Overall activity impairment due to problem, n**	423	72	144	52	43	33	79	<0.0001	DE: <0.0001 US: <0.0001	Overall 0.0294 DE: 0.0083 UK: 0.0193	<0.0001			
Mean (SD)	47.3 (24.1)	51.5 (27.1)	54.9 (19.5)	48.3 (23.2)	47.9 (23.2)	42.4 (18.6)	30.6 (24.3)							

* p values that are statistically significant are shown only in columns 10 and 11.

^†^ Patients who were employed and completed the WPAI.

^‡^ FR: EQ-5D utility score, MID (worse) vs. UK and US, MID (better) vs. ES; EQ VAS, MID (worse) vs. UK and US.

^§^ DE: EQ-5D utility score, MID (worse) vs. UK and US, MID (better) vs. ES; EQ VAS, MID (worse) vs. UK and US.

^ǁ^ IT: EQ-5D utility score, MID (worse) vs UK and US, MID (better) vs ES; EQ VAS, MID (worse) vs US.

^¶^ ES: EQ-5D utility score, MID (worse) vs FR, DE, IT, UK and US; EQ VAS, MID (worse) vs UK and US.

** UK: EQ-5D utility score, MID (better) vs FR, DE, IT and ES; EQ VAS, MID (worse) vs US; MID (better) vs. FR, DE and ES.

^††^ US: EQ-5D utility score, MID (better) vs FR, DE, IT and ES; EQ VAS, MID (better) vs FR, DE, IT, ES and UK.

EORTC QLQ-C30: For the functioning scales and global QoL, higher scores indicate better functioning; For the symptom scales and financial difficulties, lower scores indicate lower symptom burden and fewer financial difficulties, respectively.

EQ-5D-5L: Index total score and domain scores range from 0.00 to 1.00, where higher scores indicate better QoL.

MID: An EQ-5D utility score that differs by ≥0.08 from the score of another country, and an EQ VAS score that differs by of ≥7 from the score of another country are considered to have met the threshold for a MID.

WPAI: Outcomes are expressed as impairment percentages, with higher numbers indicating greater impairment and less productivity.

AN, ANOVA; DE, Germany; DLBCL, Diffuse large B-cell lymphoma; EORTC QLQ-C30, European Organisation for the Research and Treatment of Cancer Quality of Life Questionnaire Core 30; ES, Spain; EQ-5D-5L, EuroQol 5-Dimention-5 Level; QoL, quality of life; SD, standard deviation; Europe, DE/ES/FR/IT/UK; FR, France; IT, Italy; MID, minimally important difference; TT, t test; UK, United Kingdom; US, United States; VAS, visual analogue scale; WPAI, Work Productivity and Activity Impairment questionnaire.

#### 3.3.2 EORTC DLBCL DSP mean scores across lines of therapy

Relative to patients on 1L therapy, mean EORTC QLQ-C30 GHS/HRQoL (56.7 to 49.9), physical functioning (76.6 to 64.0), role functioning (62.8 to 55.3), cognitive functioning (76.3 to 70.9) and social functioning (64.6 to 58.6) scale scores were lower in patients on 3L+ therapy (**Table 5**). Only the emotional functioning score only did not significantly change when compared across all therapy lines, patients on 3L+ treatment scored lower in fatigue (40.4 to 47.1), dyspnea (22.2 to 33.9) and diarrhea (8.1 to 15.1) scales than patients on 1L treatment; differences across all therapy lines and between 1L/2L and 3L+ therapies were found for these three symptoms. Nausea and vomiting, pain, and financial difficulties also differed when scores were compared across all therapy lines, with worse scores observed in 3L+.

**Table 5:** Disease burden of patients with DLBCL, by line of therapy: bivariate analysis.

	Line of Therapy				Comparison between all lines p value*	Comparison 1L/2L vs 3L+ p value^†^
	Total N=441	1 n=87	2 n=228	3+ n=126		
					(AN)	(TT)
EORTC QLQ-C30						
**Global health status, n**	441	87	228	126	0.0119	0.0033
Mean (SD)	54.1 (19.1)	56.7 (18.1)	55.5 (18.0)	49.9 (21.1)		
**Physical functioning, n**	441	87	228	126	<0.0001	<0.0001
Mean (SD)	71.7 (21.6)	76.6 (19.7)	74.2 (20.2)	64.0 (23.3)		
**Role functioning, n**	441	87	228	126	0.0086	0.0024
Mean (SD)	61.7 (28.0)	62.8 (27.1)	64.8 (27.4)	55.3 (29.0)		
**Emotional functioning, n**	441	87	228	126	0.3470	0.4480
Mean (SD)	67.7 (23.1)	65.6 (23.5)	69.2 (22.9)	66.4 (23.0)		
**Cognitive functioning, n**	441	87	228	126	0.0172	0.0057
Mean (SD)	75.8 (23.5)	76.3 (23.8)	78.3 (23.5)	70.9 (22.7)		
**Social functioning, n**	440	87	227	126	0.0139	0.0052
Mean (SD)	64.4 (27.6)	64.6 (26.9)	67.5 (26.2)	58.6 (29.9)		
**Fatigue, n**	441	87	228	126	0.0061	0.0016
Mean (SD)	41.5 (23.3)	40.4 (22.2)	38.9 (22.9)	47.1 (25.4)		
**Nausea & vomiting, n**	441	87	228	126	0.0721	0.0334
Mean (SD)	20.0 (22.2)	20.3 (24.0)	17.9 (20.3)	23.5 (23.9)		
**Pain, n**	441	87	228	126	0.0717	0.0331
Mean (SD)	27.3 (24.8)	27.6 (24.6)	24.9 (24.5)	31.2 (25.0)		
**Dyspnea, n**	441	87	228	126	<0.0001	<0.0001
Mean (SD)	25.3 (25.2)	22.2 (25.3)	21.8 (23.0)	33.9 (27.0)		
**Insomnia, n**	439	87	227	125	0.3402	0.1831
Mean (SD)	30.3 (27.5)	31.4 (28.5)	28.5 (26.6)	32.8 (28.4)		
**Appetite loss, n**	440	87	227	126	0.4060	0.2293
Mean (SD)	35.3 (27.9)	33.7 (26.8)	34.4 (27.4)	38.1 (29.4)		
**Constipation, n)**	439	87	227	125	0.0949	0.0535
Mean (SD)	17.6 (24.4)	18.4 (26.8)	15.3 (23.3)	21.2 (24.4)		
**Diarrhea, n**	439	87	227	125	0.0260	0.0484
Mean (SD)	12.3 (18.7)	8.1 (14.4)	12.4 (18.7)	15.1 (20.9)		
**Financial difficulties, n**	439	87	227	125	0.0982	0.0327
Mean (SD)	17.0 (24.2)	16.1 (23.3)	15.2 (23.3)	20.9 (26.2)		
EQ-5D-5L						
**EQ-5D Utility score (UK crosswalk), n**	439	86	227	126	0.0149	0.0044
Mean (SD)	0.70 (0.20)	0.73 (0.19)	0.72 (0.19)	0.66 (0.23)		
**EQ VAS, n**	381	77	196	108	0.0821	0.0263
Mean (SD)	64.6 (17.9)	66.3 (16.3)	65.7 (17.0)	61.4 (20.3)		
WPAI^‡^						
**% Work time missed due to problem, n**	66	15	41	10	0.9644	0.8041
Mean (SD)	28.4 (36.0)	29.7 (39.6)	28.5 (34.6)	25.7 (40.0)		
**% Impairment while working due to problem, n**	65	16	41	8	0.2157	0.088
Mean (SD)	30.3 (31.5)	35.6 (31.4)	31.7 (32.9)	12.5 (19.1)		
**% Overall work impairment due to problem, n**	55	12	35	8	0.3338	0.137
Mean (SD)	31.1 (27.5)	33.8 (27.7)	33.2 (28.3)	17.7 (22.4)		
**% Overall activity impairment due to problem, n**	423	83	218	122	0.0022	0.0005
Mean (SD)	47.3 (24.1)	44.2 (23.3)	44.9 (23.5)	53.7 (24.8)		

* Bivariate analysis: ANOVA.

^†^ Bivariate analysis: t test.

^‡^ Patients who were employed and completed the WPAI.

EORTC QLQ-C30: for the functioning scales and global QoL, higher scores indicate better functioning; For the symptom scales and financial difficulties, lower scores indicate better symptom burden and fewer financial difficulties, respectively.

EQ-5D-5L: index total score and domain scores range from 0.00 to 1.00, where higher scores indicate better QoL.

WPAI: outcomes are expressed as percentages of work time impaired due to disease, with higher numbers indicating greater impairment and less productivity.

AN, ANOVA; DLBCL, diffuse large B-cell lymphoma; EORTC QLQ-C30, European Organisation for the Research and Treatment of Cancer Quality of Life Questionnaire Core 30; EQ-5D-5L, EuroQol 5-Dimention-5 Level; IQR, interquartile range; SD, standard deviation; TT, t-test, VAS, visual analogue scale; WPAI, Work Productivity and Activity Impairment questionnaire.

#### 3.3.3 EORTC DLBCL DSP mean scores across country

The mean (SD) GHS/HRQoL score in the US was 67.5 (19.7) and in Europe ranged from 44.4 (14.9) in the UK to 59.3 (16.2) in Italy (**Table 4**). Analysis by region showed that DLBCL patients in the US had higher scores for all EORTC QLQ-C30 functioning scales and lower scores for all symptom scales than patients in European countries. The exception was for ‘financial difficulties’ where France had the lowest score of 10.4 (20.9). In Europe, functioning tended to be lowest and symptom scale scores tended to be highest for patients in Spain, whereas the opposite was true for patients in Italy. This differed significantly from the other European countries.

#### 3.3.4 EQ-5D-5L and EQ VAS scores across lines of therapy

The mean (SD) EQ-5D-5L utility score (UK crosswalk) was 0.70 (0.20) and was reduced from 0.73 (0.19) for patients on 1L therapy to 0.66 (0.23) for patients on 3L+ (**Table 5**). The reductions from 1L to 3L+ in EQ-5D utility scores were reduced when comparing all three lines of therapy (1L vs. 2L vs. 3L) and between lines of therapy. Overall, mean (SD) EQ VAS score was 64.6 (17.9) and was lower in patients on 3L+ compared to patients on 1L therapy (66.3 [16.3] vs 61.4 [20.3], respectively), with the difference between 1L/2L and 3L+ being significant.

The mean (SD) EQ-5D utility score for patients in the US was 0.80 (0.16) and in Europe ranged from 0.60 (0.20) in Spain to 0.80 (0.15) in the UK (**Table 4**). Mean (SD) VAS score for patients in the US was 79.6 (14.8) and in Europe ranged from 56.2 (18.3) in France to 69.6 (16.5) in the UK (**Table 4**). Mean EQ-5D utility and VAS scores were higher for US patients compared to European patients. Mean EQ-5D utility and VAS scores were also higher for patients in the UK compared to the other European countries.

#### 3.3.5 EQ-5D-5L and EQ VAS scores DLBCL DSP vs MID values

The UK had significantly higher EQ-5D utility scores, greater than established MIDs, than each of the other European countries, and higher EQ VAS scores for the UK than France, Germany and Spain, but lower scores than US. France, Germany and Italy each had lower EQ-5D utility scores than with the UK and US but higher than with Spain, and lower EQ VAS scores compared than the US. Spanish patients recorded lower EQ-5D utility scores than with all other European countries and the US, and lower EQ VAS scores than the UK and US.

#### 3.3.6 WPAI scores across lines of therapy and country

Results from the WPAI assessments are presented in **Table 5**. Sample sizes were low (n ≤ 10) for work scores of patients on 3L+. The mean (SD) percent work time missed due to DLBCL was 28.4% (36.0%). The mean (SD) percentage impairment while working due to DLBCL was 30.3% (31.5%) and mean (SD) percentage overall work impairment due to DLBCL was 31.1% (27.5%). For patients on 3L+ therapy, mean (SD) percentages of impairment while working and overall work impairment were lower, 12.5% (19.1%) and 17.7% (22.4%), respectively, compared with 1L therapy (35.6% [31.4%] and 33.8% [27.7%]). The differences across therapy lines and between 1L + 2L and 3L+ therapies were not statistically significant. Patients had a mean (SD) percentage overall activity impairment due to DLBCL of 47.3% (24.1%), which increased from 1L (44.2%) to 3L+ (53.7%).

Generally, base numbers of patients in each European country were too low for meaningful comparison of productivity assessment. The percentages (SD) work time missed, impairment while working, and overall work impairment due to DLBCL were 13.2% (21.6%), 12.1% (18.8%), and 19.2% (23.5%) in the US vs. 31.3% (36.9%), 45.6% (38.6%), and 36.6% (26.0%) in Germany (n=10–16). The percentage (SD) overall activity impairment due to DLBCL for patients in the US was 30.6% (24.3%) and in Europe ranged from 42.4% (18.6%) in the UK to 54.9% (19.5%) in Germany (**Table 4**). Scores indicated that patients in Europe were more impaired in their work and activity than in the US. Activity impairment scores also indicated that UK patients were less impaired and German patients were more impaired than patients in the other European countries.

### 3.4 Factors associated with PROs/HRQoL

Factors found to be associated with PROs/HRQoL for patients with DLBCL by linear regression are shown in **Table 6**. Across all EORTC QLQ-C30 functional scales (excluding physical functioning) and EQ-5D scales, only patients who relapsed after one year on therapy had a positive co-efficient, suggesting a better QoL than patients who relapsed before one year. All other factors associated with PROs/HRQoL had a negative co-efficient, including an IPI score of three or four, DLBCL subtype (‘double hit/expressors’), those who received prior ASCT, and those who received prior CAR-T cell therapy, suggesting a worse QoL.

**Table 6b.** Symptom factors associated with QoL for patients with DLBCL (statistically significant factors for PRO scores).

**Table 6 A:** Utility/functioning factors associated with QoL for patients with DLBCL (statistically significant factors for PRO scores).

PRO tool	Factor	Reference group	Co-efficient*	p value
Utility/functional Scales				
**EQ-5D-5L utility score (UK crosswalk)**	Relapse after 1 year	No relapse	0.124	0.013
	IPI score 3	IPI score 0	-0.161	0.001
	IPI score 4	IPI score 0	-0.189	<0.001
	ASCT received	No ASCT received	-0.098	0.017
**EORTC QLQ-C30 physical functioning**	IPI score 3	IPI score 0	-15.647	<0.001
	IPI score 4	IPI score 0	-21.073	<0.001
**EORTC QLQ-C30 role functioning**	Relapse after 1 year	No relapse	14.357	0.011
	IPI score 3	IPI score 0	-20.433	<0.001
	IPI score 4	IPI score 0	-25.984	<0.001
**EORTC QLQ-C30 emotional functioning**	Relapse after 1 year	No relapse	15.704	0.002
	ASCT received	No ASCT received	-8.411	0.041
**EORTC QLQ-C30 cognitive functioning**	Relapse after 1 year	No relapse	14.562	0.004
	CAR-T cell therapy	No CAR-T cell therapy	-13.501	0.038
**EORTC QLQ-C30 social functioning**	Relapse after 1 year	No relapse	13.268	0.025
**EQ VAS**	Relapse after 1 year	No relapse	9.241	0.017
	IPI score 3	IPI score 0	-7.645	0.049
	IPI score 4	IPI score 0	-11.236	0.006
	CAR-T cell therapy	No CAR-T cell therapy	-12.712	0.021
	Triple hit^	Double hit	-9.155	0.03
	Double expressor^^	Double hit	-11.314	0.004

* A positive co-efficient indicates better utility/functioning thus a better quality of life.

^Triple hit; a patient has mutations in genes MYC, BCL-2 and BCL-6.

^^ This was captured as both double and expressor hit in the survey. Double expressor, a patient has over expression of two/three of genes MYC, BCL-2 or BCL-6.

ASCT, autologous stem cell transplant; CAR, chimeric antigen receptor; DLBCL, diffuse large B-cell lymphoma; EORTC QLQ-C30, European Organisation for the Research and Treatment of Cancer Quality of Life Questionnaire Core 30; EQ-5D, EuroQol 5-Dimention-5 Level; IPI, International Prognostic Index; QoL, Quality of Life; VAS, visual analogue scale.

**Table 6 B:** Symptom factors associated with QoL for patients with DLBCL (statistically significant factors for PRO scores).

PRO tool	Factor	Reference group	Co-efficient*	p value
Symptom scales						
**EORTC QLQ-C30 fatigue**	Relapse after 1 year	No relapse	-12.99	0.006
	IPI score 3	IPI score 0	15.465	0.001
	IPI score 4	IPI score 0	20.065	<0.001
	Double exposure	No double exposure	19.453	0.007
**EORTC QLQ-C30 nausea & vomiting**	3+ therapy lines	1-2 therapy lines	10.235	0.013
	Relapse after 1 year	No relapse	-18.36	<0.001
	IPI score 3	IPI score 0	10.33	0.026
	IPI score 4	IPI score 0	13.342	0.007
	Triple hit^	Double hit	11.314	0.033
**EORTC QLQ-C30 pain**	Relapse after 1 year	No relapse	-12.548	0.016
	IPI score 2	IPI score 0	10.538	0.028
	IPI score 3	IPI score 0	11.08	0.029
	IPI score 4	IPI score 0	21.307	<0.001
**EORTC QLQ-C30 dyspnea**	3+ therapy lines	1-2 therapy lines	9.96	0.026
	IPI score 1	IPI score 0	11.294	0.021
	IPI score 2	IPI score 0	11.612	0.015
	IPI score 3	IPI score 0	14.833	0.003
	IPI score 4	IPI score 0	21.564	<0.001
**EORTC QLQ-C30 insomnia**	3+ therapy lines	1-2 therapy lines	11.387	0.028
	Relapse after 1 year	No relapse	-18.176	0.002
	IPI score 3	IPI score 0	13.692	0.019
	IPI score 4	IPI score 0	14.864	0.017
**EORTC QLQ-C30 appetite loss**	IPI score 3	IPI score 0	11.696	0.048
	IPI score 4	IPI score 0	13.239	0.037
**EORTC QLQ-C30 constipation**	3+ therapy lines	1-2 therapy lines	10.667	0.02
	Relapse after 1 year	No relapse	-15.298	0.004
**EORTC QLQ-C30 diarrhea**	Relapse within 1 year	No relapse	5.294	0.033
**EORTC QLQ-C30 financial difficulties**	3+ therapy lines	1-2 therapy lines	11.7	0.01
	Relapse after 1 year	No relapse	-10.465	0.045
**WPAI activity impairment**	IPI score 2	IPI score 0	9.853	0.028
	IPI score 3	IPI score 0	26.706	<0.001
	IPI score 4	IPI score 0	24.801	<0.001

*A positive co-efficient indicates worse symptom scores thus a worse quality of life.

^Triple hit; a patient has mutations in genes MYC, BCL-2 and BCL-6.

Double exposure, a patient was relapse/refractory to at least two prior lines of systemic therapy, including an anti-CD20 antibody and an alkylating agent.

DLBCL, diffuse large B-cell lymphoma; EORTC QLQ-C30, European Organisation for the Research and Treatment of Cancer Quality of Life Questionnaire Core 30; IPI, International Prognostic Index; PRO, patient-reported outcomes; QoL, Quality of Life; WPAI, Work Productivity and Activity Impairment questionnaire.

Similarly, across the majority of EORTC QLQ-C30 symptom scales, only patients who relapsed after one year on therapy had a negative co-efficient, suggesting a better QoL than patients who relapsed before one year. All other factors associated with PROs/HRQoL had a positive co-efficient, including an IPI of 2, 3 or 4, DLBCL subtype (‘double hit’ lymphoma), patients on 3L+ therapy, and patients who were R/R on at least two prior lines of systemic therapy, one exception was diarrhea in patients who relapsed after one year on therapy. These patients had a positive coefficient, suggesting worse HRQoL.

### 3.5 Physician- and patient-reported symptom burden

**Figure 1:**
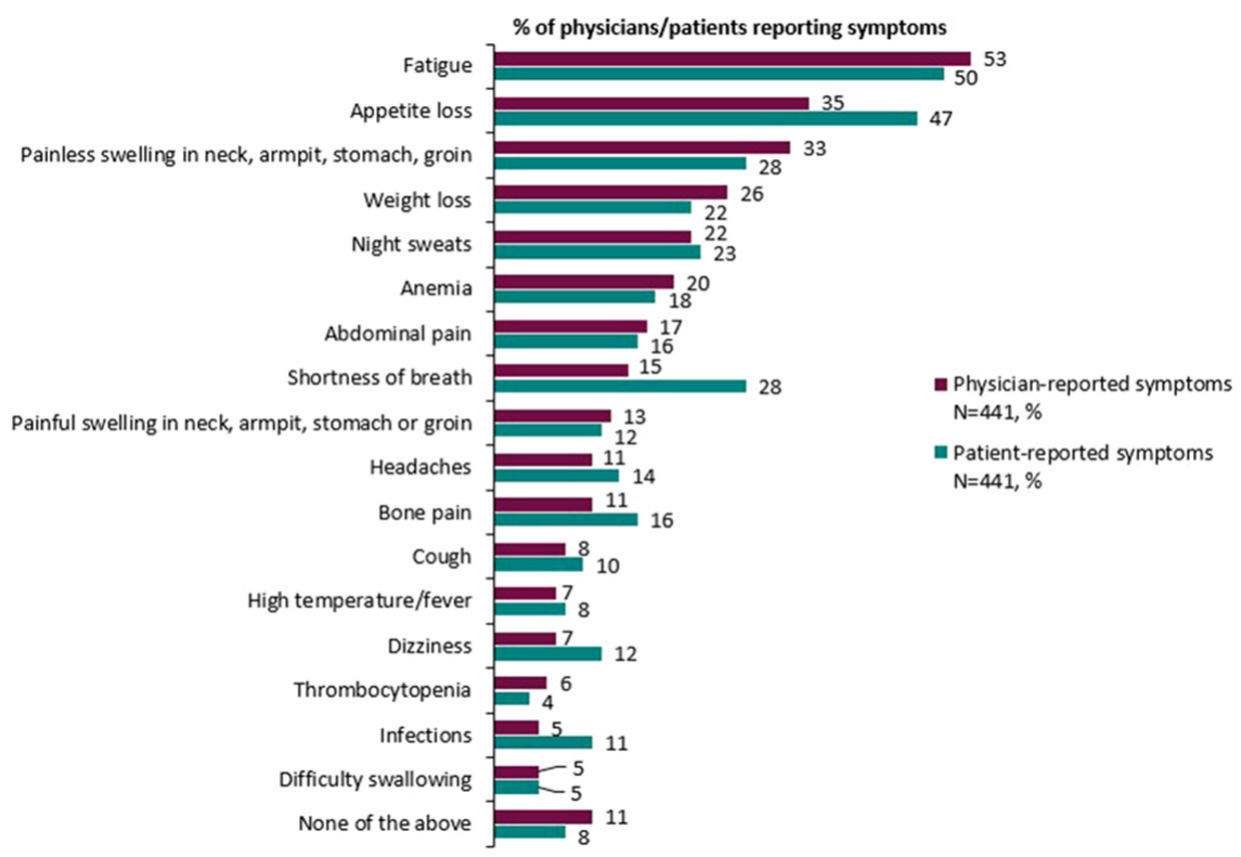
Overall physician- and patient-reported symptoms experienced by patients with DLBCL.

Broadly speaking, physician and patient reports were consistent when reporting symptoms experienced. Overall, physicians reported that 89% of patients experienced symptoms compared with 92% of patients who reported experiencing symptoms associated with DLBCL (**Table 7**). Physicians (for n=441 patients) reported that the most common symptoms experienced by patients with DLBCL were fatigue (53%), loss of appetite (35%), and painless swelling in neck/armpit/stomach/groin (33%). The proportion of matched patients (n=441) reporting that they experienced fatigue was 50%, loss of appetite was 47%, painless swelling in the neck/armpit/stomach/groin was 28%, and shortness of breath was 28%. Physician and patient reports of abdominal pain (17% and 16% respectively), high temperature and fever (7% and 8% respectively) and night sweats (22% and 23% respectively) further describe the consistency across physician- and patient-reported symptoms (**Figure 1**).

**Table 7:** Symptom burden of patients with DLBCL, by line of therapy.

Symptom at data collection (≥10% present at any one line) n (%)	Lines of Therapy									
	Physician-reported symptoms					Patient-reported symptoms				
	Total N=441	1 n=87	2 n=228	3+ n=126	p value (CH)	Total N=441	1 n=87	2 n=228	3+ n=126	p value (CH)
Abdominal pain	73 (17)	6 (7)	34 (15)	33 (26)	0.0006	72 (16)	10 (11)	34 (15)	28 (22)	0.0619
Anemia	90 (20)	13 (15)	43 (19)	34 (27)	0.071	80 (18)	14 (16)	42 (18)	24 (19)	0.8316
Bone pain	49 (11)	6 (7)	23 (10)	20 (16)	0.0954	71 (16)	11 (13)	35 (15)	25 (20)	0.3134
Cough	34 (8)	4 (5)	16 (7)	14 (11)	0.1839	44 (10)	10 (11)	19 (8)	15 (12)	0.4749
Difficulty swallowing	23 (5)	3 (3)	7 (3)	13 (10)	0.0095	23 (5)	3 (3)	13 (6)	7 (6)	0.7039
Dizziness	30 (7)	0 (0)	17 (7)	13 (10)	0.0113	52 (12)	6 (7)	29 (13)	17 (13)	0.2708
Fatigue	235 (53)	35 (40)	122 (54)	78 (62)	0.0077	221 (50)	38 (44)	111 (49)	72 (57)	0.0982
Headaches	49 (11)	3 (3)	21 (9)	25 (20)	0.0004	61 (14)	7 (8)	24 (11)	30 (24)	0.0004
High temperature/fever	32 (7)	3 (3)	16 (7)	13 (10)	0.1613	36 (8)	7 (8)	18 (8)	11 (9)	0.9529
Infections	23 (5)	1 (1)	14 (6)	8 (6)	0.1627	50 (11)	4 (5)	29 (13)	17 (13)	0.0807
Appetite loss	155 (35)	26 (30)	81 (36)	48 (38)	0.4603	208 (47)	41 (47)	105 (46)	62 (49)	0.7565
Night sweats	98 (22)	11 (13)	50 (22)	37 (29)	0.0154	103 (23)	19 (22)	48 (21)	36 (29)	0.2316
Painful swelling in neck, armpit, stomach or groin	58 (13)	6 (7)	27 (12)	25 (20)	0.0161	53 (12)	10 (11)	25 (11)	18 (14)	0.6204
Painless swelling in neck, armpit, stomach, groin	147 (33)	23 (26)	75 (33)	49 (39)	0.1627	123 (28)	26 (30)	58 (25)	39 (31)	0.4571
Shortness of breath	67 (15)	6 (7)	30 (13)	31 (25)	0.0009	123 (28)	14 (16)	60 (26)	49 (39)	0.0006
Thrombocytopenia	28 (6)	3 (3)	13 (6)	12 (10)	0.1714	17 (4)	1 (1)	10 (4)	6 (5)	0.3326
Weight loss	114 (26)	15 (17)	52 (23)	47 (37)	0.0014	97 (22)	17 (20)	48 (21)	32 (25)	0.4103
None of the above	48 (11)	19 (22)	18 (8)	11 (9)	0.0012	36 (8)	12 (14)	15 (7)	9 (7)	0.1023

CH, chi square test; DLBCL, diffuse large B-cell lymphoma.

Generally, the proportion of patients reporting symptoms was higher at later treatment lines, although the magnitude of differences varied considerably. The proportion of physicians reporting patients with symptoms across therapy lines tended to be greater than the proportion of patients self-reporting symptoms. The proportion of physicians reporting that patients experienced fatigue was 40% in patients on 1L therapy compared with 62% of patients on 3L+ therapy, and the proportion of patients self-reporting fatigue non-significantly increased from 44% to 57%, respectively. The proportion of physicians reporting patients with shortness of breath increased from 7% to 25%; and the proportion of patients self-reporting this symptom increased from 16% to 39%. The proportion of physicians reporting patients with weight loss increased from 17% to 37%. There was no significant difference across treatment lines in the proportion of physicians or patients that reported appetite loss and painless swelling in the neck/armpit/stomach/groin.

### 3.6 Adverse events

Adverse events were physician-reported for 133 (30.2%) patients; the proportions of physicians reporting patients with fatigue was 74%, anemia 49%, appetite loss 44%, and nausea was 42% (**Table 8**). For those patients on 3L+ therapy, physicians reported 81%, 61%, 42% and 52% experienced fatigue, anemia, appetite loss, and nausea, respectively. Physicians reported over one-quarter of patients experiencing aches/pains, hair loss, and/or weight loss. Cytokine release syndrome was reported for 2% of patients (0%, 1%, and 6% of patients on 1L, 2L, and 3L+ therapy). Physicians reported similar proportions of patients experiencing adverse effects across therapy lines, excluding nerve damage/neuropathy that was reported for 3% of patients (0%, 1%, and 10% of patients on 1L, 2L, and 3L+ therapy). The proportion of adverse events between patients on different lines of therapy were not found to be statistically significant in any case.

**Table 8:** Adverse events experienced by patients with DLBCL, by line of therapy.

Adverse event at data collection (≥10% present at any at any one line) n (%)	Lines of Therapy					
	Total (Patients that experienced adverse events) N=133	1 n=26	2 n=76	3+ n=31	p value (CH)
Aches and pains	42 (32)	7 (27)	23 (30)	12 (39)	0.5912
Anemia	65 (49)	13 (50)	33 (43)	19 (61)	0.2429
Chills	9 (7)	0 (0)	6 (8)	3 (10)	0.2928
Constipation	24 (18)	2 (8)	13 (17)	9 (29)	0.1075
Diarrhea	29 (22)	6 (23)	18 (24)	5 (16)	0.6812
Difficulty in breathing	11 (8)	1 (4)	6 (8)	4 (13)	0.458
Dizziness	16 (12)	1 (4)	10 (13)	5 (16)	0.3281
Fatigue	99 (74)	17 (65)	57 (75)	25 (81)	0.4147
Febrile neutropenia	22 (17)	2 (8)	13 (17)	7 (23)	0.3149
Fever/flu-like symptoms	13 (10)	1 (4)	7 (9)	5 (16)	0.289
Hair loss	39 (29)	8 (31)	23 (30)	8 (26)	0.8853
Headaches	20 (15)	1 (4)	13 (17)	6 (19)	0.1964
High blood pressure	10 (8)	1 (4)	5 (7)	4 (13)	0.388
Infections	21 (16)	2 (8)	13 (17)	6 (19)	0.4323
Insomnia	14 (11)	2 (8)	9 (12)	3 (10)	0.8248
Appetite loss	58 (44)	11 (42)	34 (45)	13 (42)	0.9548
Mood changes	12 (9)	1 (4)	6 (8)	5 (16)	0.2377
Mouth sores	17 (13)	2 (8)	10 (13)	5 (16)	0.6296
Mucositis	23 (17)	7 (27)	9 (12)	7 (23)	0.1444
Nausea	56 (42)	14 (54)	26 (34)	16 (52)	0.1021
Nerve damage/neuropathy	4 (3)	0 (0)	1 (1)	3 (10)	0.0433
Shortness of breath	12 (9)	0 (0)	7 (9)	5 (16)	0.106
Thrombocytopenia	20 (15)	1 (4)	12 (16)	7 (23)	0.1379
Vomiting	25 (19)	8 (31)	12 (16)	5 (16)	0.2191
Weight loss	36 (27)	6 (23)	21 (28)	9 (29)	0.467

CH, chi square test; DLBCL, diffuse large B-cell lymphoma.

## 4 Discussion

This real-world analysis provides a multinational survey of disease burden and PRO data from patients with DLBCL receiving 1L, 2L and 3L+ therapies. Patients with DLBCL generally presented with impairments in functioning and experienced a high symptom burden across all lines of therapy, while disease burden was significantly worse when compared with an NHL reference population with regards to PROs and symptoms. In addition, GHS/HRQoL was also worse for patients with DLBCL on 3L+ therapy than those on earlier lines of therapy, and patients on later lines of therapy experienced worse HRQoL in most functioning and symptom domains such as fatigue, dyspnea and diarrhea. These findings highlight the symptom burden and impairment in HRQoL experienced by patients with DLBCL, particularly in the 3L+ setting and highlight the unmet need for novel treatment options and better supportive care interventions to improve or slow the deterioration of GHS/HRQoL.

This analysis demonstrated that the HRQoL of patients with DLBCL varied compared to NHL reference values. GHS/HRQoL was worse for the DLBCL population but role, cognitive and social functioning were better than for the NHL reference population. Importantly, the DLBCL population experienced worse nausea and vomiting, pain, dyspnea, appetite loss, and diarrhea when compared with the NHL reference population. The findings from our DLBCL survey demonstrated that patients with DLBCL experienced worse GHS/HRQoL and a higher burden of certain symptoms than the NHL reference population, which is particularly salient given prior work demonstrating worse burden than individuals without NHL (25). A review of studies of HRQoL in patients with NHL found that the domains of HRQoL were negatively affected to varying extents in NHL survivors compared with normative populations, but the clinically important differences (according to study-defined changes in scores) for patients were physical functioning, vitality, appetite loss, and financial problems (43). Clinically important differences in GHS/HRQoL, role-physical functioning, and social functioning were infrequently reported to begin with, and have further decreased over time (43). Patients with NHL had clinically worse scores on EORTC QLQ-C30 GHS/HRQoL, physical, role, and social function, and more appetite loss before treatment compared with the age- and sex-matched normative population (44). More recently, data from the RT3 (*Real-Time Tailored Therapy*) study of DLBCL patients 2 years post-diagnosis found that patients had a similar overall EORTC QLQ-C30 GHS/QoL score but scores on physical, role, cognitive, and social functioning, as well as symptom scores of fatigue and dyspnea were marginally worse, within the limits of clinically important differences, compared with a French normative population (45). Since the data in this study was collected in 2021, differences in HRQoL of patients with DLBCL and the NHL reference values may be attributed to changes in treatment and its impact to HRQoL since 2008 when the NHL reference values were published. Another factor that may attribute to the differences between the two populations is that the present study looked solely into the HRQoL of patients with DLBCL, whereas the NHL reference values are based on patients with NHL. The reference values also do not specify what subtypes of NHL are included within their results. Further research should look to establish HRQoL of patients with DLBCL specifically.

To our knowledge, no previous real-world studies have investigated PROs in patients with DLBCL and made comparisons between Europe and the US. PRO measures of functioning, symptoms, work productivity and activity were better for patients in the US than for those in the individual European countries and for all European countries sampled. PROs varied across Europe, with better functioning generally experienced by those in Italy and the UK, and fewer symptoms experienced by patients in Germany. EQ-5D between-country comparisons using MIDs suggested an overall better health state for patients in the UK but worse health state for those in Spain compared with other European countries. These findings are likely due in part to the different healthcare systems operating within each country, which is reflected in the patients’ health insurance type, with healthcare being mainly private in the US, public in the UK, and mainly public in Germany, Italy and Spain (France is often a mix of public/private healthcare). These different healthcare systems may result in different treatment quality and varying degrees of inequity in patient access to treatment. Moreover, aside from differing healthcare systems, each country has individual practices and cultures that may lead to differences in patients’ QoL (46). The present study demonstrates the need to assess HRQoL in country-specific studies as well as internationally.

Similarly, to our knowledge there are no real-world studies investigating the PROs of patients with DLBCL across lines of therapy. The burden of DLBCL varied across lines of therapy, with patients on 3L+ therapy showing the greatest impairment in functioning, especially in the domains of physical, role, cognitive and social functioning. While symptom burden was high across all therapy lines, fatigue and appetite loss were reported as both symptoms and adverse events for high proportions of patients, both of which can result from the disease and its treatment (47–50). Prior work in patients with relapsed/refractory DLBCL noted impairments in HRQoL at diagnosis and after all treatments (17), with decreases in HRQoL (including on the EORTC QLQ-C30) and health utility while on first and later lines of therapy (51). Our analysis demonstrated that patients with DLBCL and receiving 3L+ therapy have a particularly high symptom burden and low QoL, underscoring an unmet need to develop novel treatments and interventions to improve the QoL/symptom burden for these patients.

Similarly, we found high rates of impairment in patient’s ability to work and overall activity using the WPAI. However, there were no statistically significant differences in work impairment across all lines and between initial and later lines of therapy, likely due to low patient numbers particularly in 1L and 3L+ therapy groups. Nonetheless, work impairment was significantly worse in European patients than in US patients. Few patients in our DLBCL population continued to work, likely because the majority had stage III/IV disease, and because they were of retirement age. Prior research examining work impairment is limited, however, the present study shows a substantial impact of DLBCL on patients’ ability to work.

We also identified several factors that were associated with worse HRQoL for patients with DLBCL, including an IPI score of two, three or four, prior ASCT, prior CAR-T cell therapy, refractoriness to at least two prior lines of systemic therapy, double exposure and double hit/expressor lymphoma. Only after one year of therapy was associated with better HRQoL, as opposed to relapsing within one year of therapy. Patients’ risk according to their IPI score played a considerable role in influencing HRQoL across the majority of functioning and symptom domains. Patients who did not relapse within a year on therapy may have more indolent disease, resulting in a lower symptom burden and therefore better HRQoL. These findings, which highlight patient factors associated with a high risk for impaired HRQoL, could help clinicians and researchers to identify patients who may benefit most from interventions and therapies aimed at improving HRQoL.

This study had several strengths. Importantly, the DSP has been published and validated in multiple separate therapy/disease areas, and matched responses provide evidence from physician and patient perspectives. By using local fieldwork agencies to advise on implementation and pilot materials, the methodology acknowledges potential cultural differences in DLBCL management, preventing obscuring of these unique perspectives in global data analysis. In addition, the DSP’s non-aligned non-interventional nature prevents placebo bias and promotes natural behavior from a large representative physician and patient sample in a real-world setting. While recall bias is a common limitation of surveys, the data were collected at the time of each patient’s appointment and physicians had access to patient medical records for extraction of historical data, potentially reducing this likelihood (52).

As with any study, this research is not without its limitations. Limitations of DSP surveys have also been previously described in other real-world studies employing DSP data (20, 53, 54).

To reduce selection bias, participation to complete the survey was voluntary, and this was the next “n” consulting DLBCL patients who met the inclusion criteria; therefore recruits may not be truly representative of the overall DLBCL population. Patient questionnaires were voluntary, and therefore patient-reported data was not available for all the patients that the physicians reported on.

Whilst our regression modelling included many covariates linked to outcomes in patients with DLBCL, we were unable to control for all possible influences such as systemic treatment received at 1L. This analysis was of data collected during the COVID-19 pandemic. QoL, particularly psychosocial well-being, was affected by the COVID-19 pandemic (55, 56) and could have influenced our findings. Moreover, missing data were not imputed, therefor the base of patients for analysis could vary between variables.

Despite such limitations, this study documents the QoL and work productivity of DLBCL patients and continues to advance the work to improve the QoL of this underserved population. Future analysis should look to compare patients with prognostic factors to those without, as to aid the discussion around the severity of disease burden in patients with DLBCL.

Despite new treatments, overall patient-reported outcomes of patients with DLBCL, particularly those who have relapsed/refractory disease and are on 3L+ therapies, still remains poor and patients continue to live with considerable morbidity. Outcomes varied significantly between countries, highlighting a need to ensure uniformity of treatment and equity of access. Our data highlights an unmet need for novel therapies and interventions to help minimize or slow the deterioration of HRQoL for patients with DLBCL.

## Data availability statement

All data that support the findings of this study are the intellectual property of Adelphi Real World and so are not publicly available. Data are however available upon reasonable request and with permission of Adelphi Real World (contact jake.butcher@adelphigroup.com).

## Ethics statement

The studies involving humans were approved by Western Institutional Review Board. The studies were conducted in accordance with the local legislation and institutional requirements. The participants provided their written informed consent to participate in this study.

## Author contributions

PJ: Supervision, Writing – review & editing. AB: Writing – original draft, Visualization, Validation, Methodology, Data curation, Writing – review & editing. QM: Funding acquisition, Writing – review & editing. NM: Writing – original draft, Visualization, Validation, Methodology, Data curation, Writing – review & editing. JB: Validation, Writing – review & editing, Writing – original draft, Visualization, Methodology, Data curation. IS: Writing – review & editing, Writing – original draft, Visualization, Validation, Methodology, Data curation. SW: Writing – review & editing, Validation, Formal analysis. RM: Writing – review & editing, Validation, Formal analysis. RQ: Writing – review & editing, Funding acquisition.

## Glossary

ASCT: Autologous stem cell transplant
CAR-T: chimeric antigen receptor T-cell
DLBCL: diffuse large B-cell lymphoma
DSP: Disease Specific Programme
ECOG-PS: Eastern Cooperative Oncology Group Performance Status
EphMRA: European Pharmaceutical Marketing Research Association
EQ-5D-5L: EuroQol EQ-5D questionnaire
1L: first-line therapy
GHS: global health status
GPP3: Good Publication Practice
HITECH: Health Information Technology
HRQoL: health-related quality of life
IPI: International Prognostic Index score
MID: minimally important differences
NHL: Non-Hodgkin lymphoma
PRO: patient-reported outcomes
QoL: quality of life
2L: second-line therapy
SD: standard deviations
3L+: third-line therapy and beyond
UK: United Kingdom
US: United States
VAS: visual analogue score
WPAI: Work Productivity and Activity Impairment questionnaire.
